# High Prevalence of the K65R Mutation in HIV-1 Subtype C Infected Patients Failing Tenofovir-Based First-Line Regimens in South Africa

**DOI:** 10.1371/journal.pone.0118145

**Published:** 2015-02-06

**Authors:** Lindiwe Skhosana, Kim Steegen, Michelle Bronze, Azwidowi Lukhwareni, Esrom Letsoalo, Maria A. Papathanasopoulos, Sergio C. Carmona, Wendy S. Stevens

**Affiliations:** 1 Department of Haematology and Molecular Medicine, Faculty of Health Sciences, University of the Witwatersrand, Johannesburg, South Africa; 2 National Health Laboratory Services, Charlotte Maxeke Johannesburg Academic Hospital, Johannesburg, South Africa; Institute of Molecular Genetics IMG-CNR, ITALY

## Abstract

**Background:**

Tenofovir (TDF) has replaced stavudine (d4T) as the preferred nucleoside reverse transcriptase inhibitor (NRTI) in first-line regimens in South Africa, but limited information is available on the resistance patterns that develop after the introduction of TDF. This study investigated the antiretroviral drug resistance patterns in South African HIV-1 subtype C-infected patients failing stavudine- (d4T) and tenofovir- (TDF) based first-line regimens and assess the suitability of TDF as the preferred first-line nucleotide reverse transcriptase inhibitor (NRTI).

**Methods:**

Resistance patterns of HIV-1 from 160 adult patients virologically failing TDF- (n = 80) and d4T- (n = 80) based first-line regimens were retrospectively analyzed. The *pol* gene was sequenced using an in-house protocol and mutations were analysed using the IAS-USA 2014 Drug Resistance Mutation list.

**Results:**

Compared to d4T-exposed patients (n = 7), patients failing on a TDF-containing regimen (n = 43) were almost 5 times more likely to present with a K65R mutation (aRR 4.86 95% CI 2.29 – 10.34). Y115F was absent in the d4T group, and detected in 13.8% (n = 11) of TDF-exposed patients, p = 0.0007. Virus from 9 of the 11 patients (82.0%) who developed the Y115F mutation also developed K65R. Intermediate or high-level resistance to most NRTIs was common in the TDF-treatment group, but these patients twice more likely to remain susceptible to AZT as compared to those exposed to d4T (aRR 2.09 95% CI 1.13 – 3.90).

**Conclusion:**

The frequency of the TDF induced K65R mutation was higher in our setting compared to non-subtype C dominated countries. However, despite the higher frequency of cross-resistance to NRTIs, most patients remained susceptible to AZT, which is reflected in the South African treatment guidelines that recommend AZT as an essential component of second-line regimens.

## Introduction

At the end of 2012 there were approximately 2.1 million people in South Africa on antiretroviral treatment (ART) [1]. Under normal circumstances antiretroviral drugs curb viral replication, but factors such as poor drug absorption; drug toxicity and intolerability can lead to low levels of adherence [2,3], resulting in subsequent development of drug resistance-associated mutations which interfere with the way in which ART functions [4].

Before 2010, the widely used first-line regimen in South Africa consisted of stavudine (d4T) and lamivudine (3TC) in combination with efavirenz (EFV) or nevirapine (NVP) [5]. Due to its toxicity and global concerns around drug safety, the 2010 South African treatment guidelines stipulated that d4T be phased out and replaced by tenofovir (TDF) [6]. Thus, this drug is fairly new to the South African roll-out program and little is known about possible drug resistance profiles arising when the drug is introduced into this setting. Thus far, only three South African studies have investigated the antiretroviral drug resistance mutation patterns in patients treated with TDF. One study demonstrated that more than 65% of the TDF exposed patients developed the K65R mutation [7], whereas the second study found K65R to be prevalent in 46% of the patients treated with TDF [8]. A third study showed a K65R prevalence of 23%, which is substantially lower compared to the other two studies [9]. Of particular concern, the K65R mutation leads to cross-resistance to most of the nucleoside reverse transcriptase inhibitors (NRTIs) [10] and therefore jeopardizes the effectiveness of second line regimens.

This retrospective study compares HIV-1 antiretroviral drug resistance patterns in patients failing d4T- and TDF-based first-line regimens, assesses the suitability of TDF as the preferred first-line NRTI, and evaluates the impact of these resistance patterns on subsequent 2^nd^ line regimens.

## Methods

### Study design and patients

HIV drug resistance results obtained between January 2010 and August 2012 from adult patients failing ART were retrieved from the database at the HIV Genotyping Laboratory at Charlotte Maxeke Johannesburg Academic Hospital (CMJAH), South Africa. Only patients on d4T- (n = 80) or TDF-based (n = 80) first-line regimens with at least one resistance mutation were randomly selected and included in this study. Demographic and clinical data collected from laboratory request forms or the laboratory information system (LIS) included age, gender, viral load, CD4 cell count, antiretroviral drug treatment regimen and duration on failing treatment.

### Pol genotyping and resistance mutation analysis

Viral RNA was extracted from 500μl of plasma using the automated EasyMag system (Biomerieux, Marcy I’Etoile, France) according to manufacturer’s instructions. The *reverse transcriptase* and *protease* regions of the virus were amplified using an in-house resistance testing assay as previously described [11,12]. Samples that could not be amplified using the in-house assay were amplified using the ViroSeq HIV-1 Genotyping System Version 2.0 (Celera Diagnostics, Alameda, CA, USA) according to manufacturer’s instructions. The lower limit of detection of all genotyping methods was 1000 RNA copies/ml. Sequences were manually edited using Sequencher version 4.8 (Genecodes, MI, USA) or ViroSeq version 2.8 software (Celera Diagnostics, Alameda, CA, USA) and subsequently submitted to the ViroScore database (Advanced Biological Laboratories (ABL), South Africa) which used the Stanford HIV database version 7.0 for the identification of the HIV-1 drug resistance mutations based on the International AIDS Society 2014 mutation list [13]. The resistance profile was categorized according to the level of resistance conferred by the mutations. Three main categories are used in this analysis: high-level resistance (R), intermediate resistance (I, combines intermediate and low-level resistance) and total susceptibility (S, combines susceptible and potential low-level resistance calls). Subtypes were determined using the REGA subtyping tool v2.0 [14]. Mega version 5 software [15] was used to construct a phylogenetic tree to ensure that no longitudinal patient samples were included in the analysis.

### Sequence Data

The *pol* nucleotide sequences were submitted to GenBank using Sequin v9.50 (www.ncbi.nlm.nih.gov/Sequin) and are available under accession numbers KM115717 to KM115876.

### Statistical analysis

GraphPad software (GraphPad Inc, USA) was used to perform Fischer’s exact test. A p-value of <0.05 was considered statistically significant. Log-binomial regression analysis was used to test the association between different mutations and NRTI (TDF vs. d4T) or NNRT (EFV vs. NVP) pressure. The relative risk (RR) and corresponding 95% confidence intervals (CI) were calculated. Mutations identified as being significantly associated with drug pressure in the univariate analysis (p<0.2) and priori variables of importance were included in multivariate models. Models were adjusted (aRR) for CD4 count, viral load and time on ART where possible, which is limited by the number of observations in each cell after stratification.

### Ethics Statement

This study was conducted with Ethical clearance by the Research on Human Subjects (Medical) Committee at the University of the Witwatersrand (Clearance Number M120730). Due to the retrospective nature of this study, no informed consent was obtained from the patients. This is in line with the Research on Human Subjects (Medical) Committee policy which states informed consent is not required for this type of study and a waiver was hence granted.

## Results

One hundred and sixty (n = 160) sequences were included for analysis. These sequences were obtained from patients failing d4T+3TC+EFV/NVP (n = 80) and patients failing TDF+3TC+EFV/NVP (n = 80). HIV-1 subtype C was the dominant subtype (99.4%, n = 159), with only one patient being infected with an unassigned subtype (0.6%) The median age of patients was 36 years, (range: 16–62) and most patients were female (n = 112, 70.0%). The median time on the failing treatment was 38 months (range: 0.5–95 months) for the patients exposed to d4T and 12 months (range: 0.5–50 months) for the TDF-exposed patients (p<0.0001). The patient demographics and clinical characteristics are summarized in Table 1.

**Table 1:** Patient demographics and clinical characteristics of South African patients failing stavudine (d4T)- or tenofovir (TDF)-based 1^st^-line regimens.

	All Patients (n = 160)			d4t-group (n = 80)			TDF-group (n = 60)			
	N (%)	Median	Range	N (%)	Median	Range	N (%)	Median	Range	p-value
Gender										
Female	112 (70.0)			52 (65.0)			60 (75.0)			0.227
Male	48 (30.0)			28 (35.0)			20 (25.0)			
Age (years)	158 (98.8)	36	16–62	80 (100)	35	19–62	78 (98.0)	36	16–59	0.920
CD4 count (cells/μl)	158 (98.8)	130	1–1007	79 (98.8)	91	1–484	79 (98.8)	165	3–1007	0.0005
VL (log_10_ RNA copies/ml)	158 (98.8)	4.7	3.2–6.6	78 (98.0)	4.7	3.6–6.5	80 (100)	4.6	3.2–6.8	<0.001
Time on current ART regimen (months)	160 (100)	21.5	0.5–95	80 (100)	12	0.5–50	80 (100)	38	0.9–95	<0.0001
Treatment										
3TC+NVP	68 (42.5)			30 (37.5)			38 (47.5)			0.263
3TC+EFV	92 (57.5)			50 (62.5)			42 (52.5)			

N: Number of patients, VL: RNA Viral Load, ART: Antiretroviral treatment, 3TC: Lamivudine, NVP: Nevirapine, EFV: Efavirenz.

**Fig 1:**
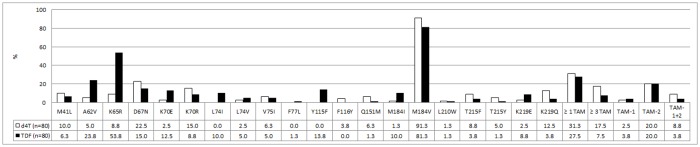
Prevalence of nucleoside reverse transcriptase inhibitor mutations and thymidine analogue mutations (TAMs) in tenofovir (TDF; black) and stavudine (d4T, white) treatment groups. The mutations that were not detected in either treatment groups are not included in the graph. The numbers represent percentages. P1: TAM pathway 1, P2: TAM pathway 2, P1+2: combination of TAM pathway 1 and 2.

The most common NRTI mutation in both the d4T- and TDF-exposed patients was M184V/I (n = 74, 92.5% and n = 72, 90.0% respectively), with M184V as the predominant mutation (Fig. 1). The K70E mutation, which is known to be selected under TDF pressure, was present in 10 (12.5%) of the TDF-exposed patients as compared to only two (2.5%) of the d4T-exposed patients (not significant). K70R was more frequently detected in d4T-exposed patients (15.0%, n = 12) as compared to the TDF-exposed group (8.8%, n = 7) but again, the difference was not significant. Compared to d4T-exposed patients (n = 7), patients failing on a TDF-containing regimen (n = 43) were almost 5 times more likely to have a K65R mutation detected (aRR 4.86 95% CI 2.29–10.34, Table 2). Y115F was absent in the d4T group, and detected in 13.8% (n = 11) of TDF-exposed patients, p = 0.0007. Nine of the 11 patients (82.0%) who developed the Y115F also developed the K65R mutation. The Q151M mutation was detected in five (6.3%) d4T-exposed patients, and in one of these patients Q151M occurred in combination with K65R. Similarly, only one (1.3%) TDF-exposed patient carried Q151M, co-existing with K65R, but this difference was not statistically significant.

**Table 2:** Crude and adjusted relative risk calculations for mutation prevalence and drug resistance call between different groups.

Mutation	N, %	Crude RR 95% CI	P value	Adjusted RR 95% CI	P value	Adjusted with
M184V/I						
d4T	74/146 (51.7%)	1.0				
TDF	72/146 (49.3%)	0.97 (0.88–1.07)	0.576	ND	ND	ND
K70E						
d4T	2/12 (16.7%)	1.0		1.0		
TDF	10/12 (83.3%)	5.0 (1.13–22.10)	0.034	2.11 (0.36–12.35)	0.409	CD4 and time on ART
K70R						
d4T	12/19 (63.2%)	1.0				
TDF	7/19 (36.8%)	0.58 (0.24–1.41)	0.230	ND	ND	ND
K65R						
d4T	7/50 (14.0%)	1.0		1.0		
TDF	43/50 (86.0%)	6.14 (2.94–12.83)	<0.0001	4.86 (2.29–10.34)	<0.0001	CD4 and VL
Q151M						
d4T	5/6 (83.3%)	1.0		1.0		
TDF	1/6 (16.7%)	0.2 (0.02–1.67)	0.138	0.13 (0.01–1.24)	0.078	CD4, VL and time on ART
≥1 TAM						
d4T	25/47 (53.2%)	1.0				
TDF	22/47 (46.8%)	0.88 (0.54–1.42)	0.603	ND	ND	ND
≥3 TAMs						
d4T	14/20 (70.0%)	1.0		1.0		
TDF	6/20 (30.0%)	0.43 (0.17–1.06)	0.066	0.53 (0.18–1.59)	0.257	CD4, VL and time on ART
TAM 1 + 2						
d4T	7/10 (70.0%)	1.0				
TDF	3/10 (30.0%)	0.43 (0.12–1.60)	0.207	ND	ND	ND
K65R + TAM						
d4T	2/8 (25.0%)	1.0				
TDF	6/8 (75.0%)	2.5 (0.50–12.5)	0.265	ND	ND	ND
K103N						
NVP	26/80 (32.5%)	1.0				
EFV	54/80 (67.5%)	1.54 (1.09–2.17)	0.016	1.43 (1.02–2.01)	0.041	CD4, VL and time on ART
V106M						
NVP	8/48 (16.7%)	1.0				
EFV	40/48 (83.3%)	3.70 (1.85–7.38)	0.0002	3.53 (1.77–7.05)	0.0004	CD4, VL and time on ART
Y181C						
NVP	32/38 (84.2%)	1.0		1.0		
EFV	6/38 (15.8%)	0.14 (0.06–0.31)	<0.0001	0.16 (0.08–0.35)	<0.0001	CD4, VL and time on ART
P225H						
NVP	3/19 (15.8%)	1.0				
EFV	16/19 (84.2%)	3.94 (1.20–13.0)	0.024	4.68 (1.43–15.37)	0.011	CD4 and VL
Predicted resistance outcome	N, %	Crude RR 95% CI	P value	Adjusted RR 95% CI	P value	Adjusted with
S TDF						
d4T	53/72 (73.6%)	1.0				
TDF	19/72 (26.4%)	0.36 (0.23–0.55)	<0.0001	0.36 (0.22–0.60)	<0.0001	CD4, VL and time on ART
IR TDF						
d4T	19/35 (54.3%)	1.0				
TDF	16/35 (45.7%)	0.84 (0.47–1.52)	0.567	ND	ND	ND
HR TDF						
d4T	8/53 (15.1%)	1.0				
TDF	45/53 (84.9%)	5.63 (2.83–11.16	<0.0001	4.41 (2.18–8.91)	<0.0001	CD4, VL
S d4T						
d4T	45/62 (72.6%)	1.0				
TDF	17/62 (27.4%)	0.38 (0.24–0.60)	<0.0001	0.40 (0.22–0.69)	0.001	CD4, VL and time on ART
IR d4T						
d4T	17/69 (24.6%)	1.0				
TDF	52/69 (75.4%)	3.06 (1.95–4.80)	<0.0001	2.83 (1.61–4.96)	0.0003	CD4, VL and time on ART
HR d4T						
d4T	18/29 (62.1%)	1.0		1.0		
TDF	11/29 (37.9%)	0.61 (0.31–1.21)	0.158	0.44 (0.21–0.96)	0.040	CD4, VL and time on ART
S ABC						
d4T	3/6 (50.0%)	1.0				
TDF	3/6 (50.0%)	1.0 (0.21–4.81)	1.000	ND	ND	ND
IR ABC						
d4T	60/85 (70.6%)	1.0		1.0		
TDF	25/85 (29.4%)	0.42 (0.29–0.59)	<0.0001	0.92 (0.81–1.03)	0.151	CD4, VL and time on ART
HR ABC						
d4T	17/69 (24.6%)	1.0		1.0		
TDF	52/69 (75.4%)	3.06 (1.94–4.80)	<0.0001	1.96 (1.06–3.61)	0.032	CD4, VL and time on ART
S ddI						
d4T	40/45 (88.9%)	1.0				
TDF	5/45 (11.1%)	0.13 (0.05–0.30)	<0.0001	0.13 (0.06–0.32)	<0.0001	VL
IR ddI						
d4T	24/44 (54.6%)	1.0				
TDF	20/44 (45.4%)	0.83 (0.50–1.38)	0.002	0.92 (0.47–1.82)	0.818	CD4, VL and time on ART
HR ddI						
d4T	16/71 (22.5%)	1.0				
TDF	55/71 (77.5%)	3.43 (2.16–5.46)	<0.0001	2.09 (1.13–3.90)	0.020	CD4, VL and time on ART
S AZT						
d4T	52/120 (43.3%)	1.0				
TDF	68/120 (56.7%)	1.31 (1.09–1.57)	0.0045	2.09 (1.13–3.90)	0.020	CD4, VL and time on ART
IR AZT						
d4T	12/19 (63.2%)	1.0				
TDF	7/19 (36.8%)	0.58 (0.24–1.41)	0.229	ND	ND	ND
HR AZT						
d4T	16/21 (76.2%)	1.0				
TDF	5/21 (23.8%)	0.32 (0.12–0.81)	0.017	0.23 (0.09–0.60)	0.003	CD4
S ETR						
d4T	39/61 (63.9%)	1.0		1.0		
TDF	22/61 (36.1%)	0.56 (0.37–0.86)	0.008	0.89 (0.71–1.11)	0.312	CD4, VL and time on ART
IR ETR						
d4T	34/82 (41.5%)	1.0		1.0		
TDF	48/82 (58.5%)	1.42 (1.03–1.93)	0.030	1.35 (0.89–2.04)	0.151	CD4, VL and time on ART
HR ETR						
d4T	7/17 (41.2%)	1.0				
TDF	10/17 (58.8%)	1.43 (0.57–3.57)	0.448	ND	ND	ND
S RPV						
d4T	26/44 (59.1%)	1.0		1.0		
TDF	18/44 (40.9%)	0.69 (0.41–1.16)	0.162	0.87 (0.63–1.21)	0.420	CD4, VL and time on ART
IR RPV						
d4T	35/73 (48.0%)	1.0				
TDF	38/73 (52.0%)	1.09 (0.77–1.52)	0.634	ND	ND	ND
HR RPV						
d4T	19/43 (44.2%)	1.0				
TDF	24/43 (55.5%)	1.26 (0.75–2.11)	0.375	ND	ND	ND

Log-binomial regression analysis was used and the model was adjusted with CD4, VL and time on treatment where possible. Adjusted relative risk was only calculated for N: Number of patients; RR: relative risk; CI: confidence interval; VL: RNA Viral Load; ART: antiretroviral treatment; 3TC: lamivudine; FTC: emtricitabine; ABC: abacavir; AZT: zidovudine; d4T: stavudine; ddI: didanosine; TDF: tenofovir; EFV: efavirenz; ETR: etravirine; NVP: nevirapine; RPV: rilpivirine; S: susceptible; IR: intermediate resistance; HR: high-level resistance; ND: not done

At least one Thymidine Analogue Mutation (TAM) was detected in 27.5% (n = 22) of the patients exposed to TDF and 31.3% (n = 25) of the d4T-exposed patients (Fig. 1). The accumulation of more than three TAMs was more common in d4T-exposed patients (n = 14, 17.5%) compared to TDF-exposed patients (n = 6, 7.5%) but the difference was not statistically significant. None of the four TDF-exposed patients who developed more than three TAMs were known to have had prior exposure to d4T. The TAM-2 pathway was the principal pathway in both groups, as 20.0% (n = 16) patients in each group followed this pathway. In three TDF-treated patients (3.8%) and seven d4T-treated patients (8.8%) a combination of both TAM-1 and TAM-2 pathways was detected (Fig. 1). Six (7.5%) TDF-exposed patients and two (2.5%) patients failing a d4T-regimen harboured the K65R mutation in combination with TAMs. All the individual TAMs, except L210W, occurred more frequently in d4T-exposed patients (Fig. 1); however the difference was not statistically significant.

**Fig 2:**
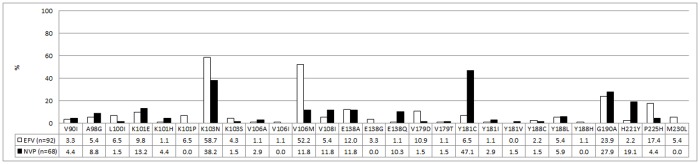
Prevalence of non-nucleoside reverse transcriptase inhibitor mutations after exposure to nevirapine (NVP) containing regimen (n = 68, black) and efavirenz (EFV) containing regimen (n = 92, white). The mutations that were not detected in either treatment groups are not included. The numbers represent percentages.

The K103N mutation was the most common non-nucleoside reverse transcriptase inhibitor (NNRTI) mutation. This mutation was more common in the EFV group (58.7%, 54 of 92) compared to the NVP group (38.2%, 26 of 68), (aRR 1.43 95% CI 1.02–2.01) (Fig. 2, Table 2). Compared to patients in the NVP group (n = 8), patients under EFV pressure (n = 40) were 3.5 times more likely to present with a V106M mutation (aRR 3.53 95% CI 1.77–7.05, Fig. 2 and Table 2). In contrast the Y181C mutation was found in 47.1% (n = 32) and 6.5% (n = 6) of the NVP and EFV group respectively (aRR 0.16 95% CI 0.08–0.35, Fig. 2 and Table 2). The frequency of the P225H mutation was significantly higher in the patients taking EFV (16 of 92, 17.4%) compared to the NVP treatment group (3 of 68, 4.4%, aRR 4.68 95% CI 1.43–15.37, Fig. 2 and Table 2).

**Fig 3:**
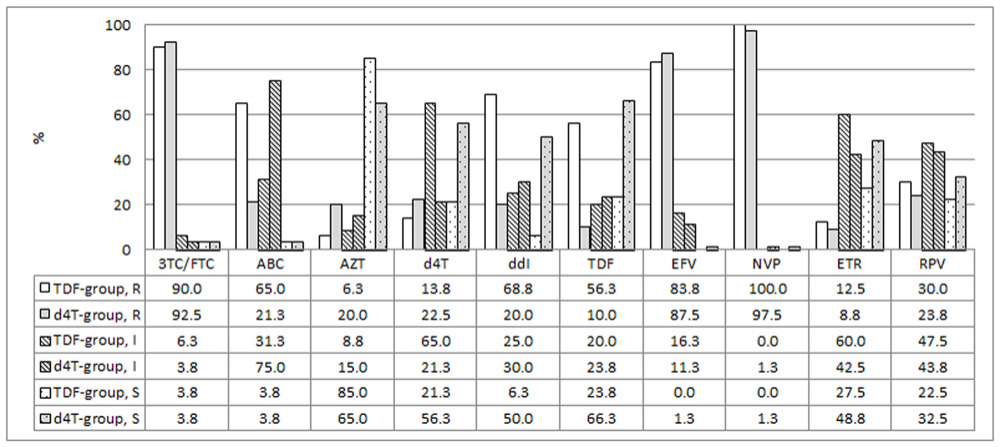
Prevalence of resistance to nucleoside/tide inhibitors (NRTI) and non- nucleoside Reverse Transcriptase Inhibitors (NNRTI). Prevalence of susceptible (S, dotted bars), intermediate (I, striped bars) or high-level resistance (R, solid bars) to NRTIs and NNRTIs after exposure to a tenofovir (TDF) containing regimen (n = 80, black) or stavudine (d4T) containing regimen (n = 80, white). The numbers represent percentages. 3TC: lamivudine; FTC: emtricitabine; ABC: abacavir; AZT: zidovudine; d4T: stavudine; ddI: didanosine; TDF: tenofovir; EFV: efavirenz; ETR: etravirine; NVP: nevirapine; RPV: rilpivirine.

The antiretroviral drug resistance profiles observed in both groups are depicted in Fig. 3. TDF exposure caused development of high-level resistance to TDF in 56.3% (n = 45) of patients, while 20.0% (n = 16) of the patients in this group showed intermediate resistance to TDF. Compared to d4T-exposed patients, those exposed to TDF were more likely to develop high-level resistance to TDF (aRR 4.41 95% CI 2.18–8.91, Table 2). TDF-exposed patients were less likely to remain fully susceptible to d4T (aRR 0.40 95% CI 0.22–0.69) and 2.8 times more likely to develop intermediate resistance to d4T (aRR 2.83, 95% CI 1.61–4.96, Table 2). Exposure to TDF increased the relative risk of developing high-level resistance to ABC (aRR 1.96 95% CI 1.06–3.61) and ddI (aRR 2.09 95% CI 1.13–3.90, Table 2) compared to exposure to d4T. High-level resistance to (AZT) was more often observed in the d4T-treatment group (20.0%, n = 16) compared to the TDF group (6.3%, n = 5), (p = 0.003). Intermediate resistance to AZT was detected in 15.0% (n = 12) of patients taking d4T and 8.8% (n = 7) of the TDF-treatment group. More importantly, patients exposed to TDF were twice more likely to remain fully susceptible to AZT as compared to those exposed to d4T (aRR 2.09 95% CI 1.13–3.90).

High-level resistance to etravirine (ETR) was observed in low numbers in both the TDF and d4T treatment groups. Only 8.8% (n = 7) of the d4T-exposed patients and 12.5% (n = 10) of the TDF patients presented with high-level resistance to ETR. Almost one in four from the d4T-exposed group (n = 19, 23.8%) and 1 in three patients in the TDF-exposed group (n = 24, 30.0%) presented with high-level resistance to RPV. Intermediate resistance to ETR and RPV was observed in 60.0% (n = 48) and 47.5% (n = 38) of the TDF-treated patients and 42.5% (n = 34) and 43.8% (n = 35) of the d4T-exposed patients.

## Discussion

In recent years, South Africa has shown significant progress in initiating HIV-infected patients on ART. However, like other African countries, South Africa has a limited number of antiretroviral drug options available; therefore it is necessary to monitor the HIV-1 drug resistance patterns that develop under the current treatment regimens, and to assess the suitability of available second line regimens. In this respect the antiretroviral drug resistance profiles among d4T-treated patients were compared with the resistance patterns developed under TDF pressure.

Since TDF is a recent addition to South African ART guidelines, the finding that duration of treatment was significantly longer in the d4T group, when compared to the TDF group, was an expected one. Although the development of TAMs has been associated with prolonged treatment failure [16], no association was found between the development of any TAMs in either treatment group, although the development of ≥ 3 TAMs which was slightly more common in the d4T-group. This finding is consistent with previous reports from South Africa, where the prevalence of ≥ 3TAMs in patients failing a d4T-based regimen was found to be between 12.0 and 17.7% [17,18]. Parikh et al. have previously shown an antagonistic association between TAMs and K65R [19]. This was confirmed by our data as only 6.2% and 1.2% of the patients, exposed to TDF and d4T respectively, developed both K65R and TAMs. More than half of the patients failing a TDF-based regimen developed the K65R mutation as compared to only 8.8% in the d4T-group, which translates into an almost 5-fold risk of developing K65R after TDF exposure. Similar results were observed by two recent South African studies [7,8], whereas the prevalence of K65R was substantially lower in a third South African study [9]. In the latter study a K65R prevalence of 23% was detected among patients presenting with at least one drug resistance mutation. The treatment duration in this study was 261 days (8.7 months) compared to 12 months in our TDF-exposed group. In addition the patients in Hoffmann’s study received more frequent viral load monitoring, which is likely to contribute to earlier detection of virological failure. A similar prevalence of K65R, compared to Hoffmann et al. was found in a Sub-Sahara African cohort where the mutation was detected in 27.7% of patients failing TDF-containing regimens and 15.0% of patients failing d4T-containing regimens [20]. A study conducted in Nigeria suggested that TDF-exposed patients infected with HIV-1 subtype G or CRF02_AG were less likely to develop K65R (14%) [18]. Similarly, K65R was only found in 17% of TDF-experienced subtype B infected patients [21]. The high frequency of K65R in our study was expected, as 98% of the study population was infected with a subtype C virus and the development of K65R is subtype dependent [22–25].

Y115F was absent in the d4T-exposed patients and detected in 13.8% of the TDF-exposed patients, which confirms the findings of a recent South African study [8]. This Y115F mutation is known to develop under ABC pressure [26], however none of the patients in our study population were exposed to ABC. *In vitro* experiments with TDF-3TC exposure showed that Y115F is commonly selected after the development of K65R [27], which is reflected in our data as K65R was present in nine out of 11 of the patients with Y115F. The sole presence of K65R causes intermediate resistance to TDF [28] whereas the combination with Y115F increases the level of resistance, as was observed in 10% of the TDF-exposed patients. Over 90% of the TDF-exposed patients had some level of resistance to three or more NRTIs. This is in contrast to what has been reported in a recent Nigerian study [29], highlighting that HIV-1 subtype is one of the factors affecting resistance and mutation patterns [30]. However, 86.3% (n = 69) of the patients failing a TDF-based regimen remained completely susceptible to AZT, which is a core component of the recommended 2^nd^-line regimen.

Our data shows that fewer TDF-exposed patients remained fully susceptible to the second generation NNRTIs ETR and RPV as compared to those patients exposed to d4T-regimens. Nonetheless, in less than 13.0% of the TDF-exposed patients, high-level resistance to these second-generation NNTRIs was detected.

This study had certain limitations, namely the fact that it was a retrospective analysis of routine laboratory data with limited clinical data and, occasionally, incomplete antiretroviral treatment history. In some cases this may have led to incorrect categorization of the patients with regards to their ART exposure. In the absence of national HIV-1 drug resistance testing guidelines, the dataset may be biased towards sampling patients under the care of clinicians reliant on HIV-1 drug resistance testing for clinical management and may not provide a proportional representation of patients failing ART in South Africa.

Overall, these results demonstrate that the frequency of the K65R mutation is higher in South African HIV-1 subtype C infected patients when compared to other countries where non-subtype C viruses dominate the epidemic. High-level resistance to NRTIs, which are the backbone of 1^st^- and 2^nd^- line treatment in South Africa, was more frequently observed after TDF-exposure compared to d4T-exposure. Despite the NRTI cross-resistance caused by K65R, most patients remained susceptible to AZT regardless of d4T or TDF-exposure. This is reflected in the South African treatment guidelines, which recommend AZT as an essential component of 2^nd^ line regimens [31]. Based on these results, TDF remains a relatively good option for first-line regimens in HIV-1 subtype C infected patients. However, the high prevalence of the K65R mutation leaves few choices to recycle NRTIs in third-line and salvage regimens.
